# Right middle lobectomy for a primary pulmonary leiomyoma: a case report

**DOI:** 10.4076/1757-1626-2-8673

**Published:** 2009-09-08

**Authors:** Michael S Vercillo, Anthony W Kim, Lisa Pitelka, Paolo Gattuso, Michael J Liptay

**Affiliations:** 1 Rush University Medical Center - University Thoracic Surgeons, 1725 W. Harrison St, Suite 774, Chicago, IL 60612, USA; 2 Rush University Medical Center - Department of Pathology, 1750 W. Harrison St, Jelke Bldg 532, Chicago, IL 60612, USA

## Abstract

**Introduction:**

Primary leiomyoma of the lung is a rare benign tumor that usually presents as a solitary lesion predominantly in young females. Fewer than 100 cases have been reported. Common symptoms include fever, chronic cough, hemoptysis, chest pain, shortness of breath, and pneumonias.

**Case presentation:**

A 34-year-old, non-smoker female who presented with recurrent pneumonias. She was found to have a primary leiomyoma of the right middle lobe. This was treated by right middle lobectomy. 6-month follow up showed patient doing well without evidence of residual disease on computerized tomography.

**Conclusion:**

Primary pulmonary leiomyoma is a rare tumor distinct from benign metastasizing leiomyoma. Histologic features include absence of mitotic count, low cellularity, lack of cytologic atypia and pleomorphism. Treatment is by conservative surgical resection and carries a favorable prognosis.

## Introduction

Primary leiomyoma of the lung is a rare benign tumor of mesodermal origin. It accounts for about 2% of all benign lung tumors [1]. Since it was first described by Forkel [2] in 1910, there have been less than 100 reported cases. We present the case of a young female who had a pulmonary leiomyoma. This case is presented because of its rarity and the paucity of literature on the tumor in the past 20 years.

## Case presentation

**Figure 1:**
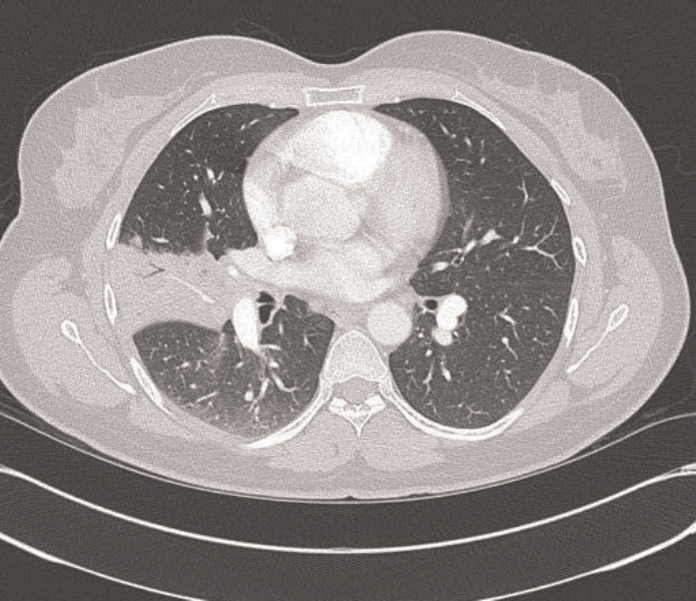
**Chest CT scan demonstrating pleural thickening of the inferior half of the right middle fissure with adjacent subsegmental atelectasis of the lateral segment of the right middle lobe with some air bronchograms and bronchiectasis seen within it**.

This case is a 34-year-old Caucasian female nonsmoker who presented with recurrent pneumonias of her right middle lobe. She experienced recurrent fevers, cough, and occasional pleuritic pain for approximately one year. She had no gynecologic issues and her pelvic exam was normal. Chest radiograph showed a bandlike density in the area of the right middle lobe. Computerized tomography (CT) of the chest showed pleural thickening of the inferior half of the right middle fissure with adjacent subsegmental atelectasis of the lateral segment of the right middle lobe with some air bronchograms and bronchiectasis seen within it (Figure 1). Bronchoscopy with biopsies revealed an obstructing lesion of the right middle lobe bronchus with fragments of tumor with interlacing fascicles of smooth muscle cells with spindled nuclei lacking atypia and mitotic activity consistent with a primary endobronchial leiomyoma.

**Figure 2:**
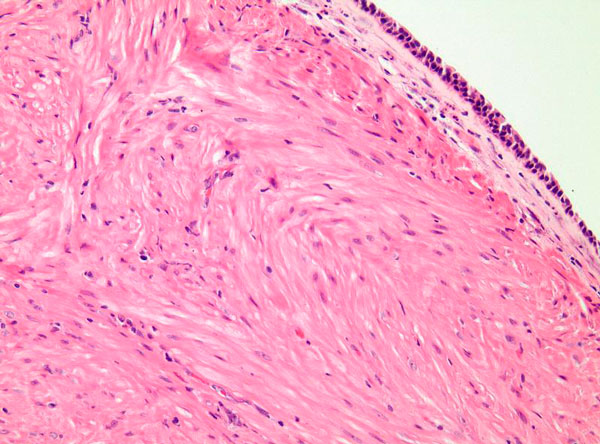
**Histopathologic slide with hematoxylin and eosin stain 20× demonstrating the abundant eosinophilic cytoplasm, oval nuclei, and inconspicuous nucleoli are appreciated**. There is no cellular atypia, no vascular invasion, and no mitotic figures.

She was taken to the operating room for a right middle lobectomy that required opening of the right middle lobe bronchus with hand-sewn bronchial closure and mediastinal lymph node sampling. The resected specimen contained a well-circumscribed mass contained within the bronchus attached by a stalk, deep to the actual bronchial margin. Pathology revealed a leiomyoma with acute and chronic inflammation and interstitial fibrosis in the remaining lung tissue (Figure 2). The tumor was strongly positive for smooth muscle actin (SMA) immunostain and negative for CD117 and S100 immunostains. The lymph nodes were all described as reactive. On 6-month follow-up, the patient's symptoms have resolved and she was doing well. CT of the chest at 3 months post-resection shows no residual disease.

## Discussion

Primary leiomyoma of the lung is a solitary lesion predominantly in females presenting in the young to middle aged, with a mean age of 35 years [3]. Common symptoms include fever, chronic cough, hemoptysis, chest pain, shortness of breath, and pneumonias. They can also be asymptomatic, found incidentally or at autopsy [4]. Histologic features include absence of mitotic count, low cellularity, lack of cytologic atypia and pleomorphism, and prominent fibrosis or hylanization [5]. The neoplastic cells are strongly and diffusely positive for SMA, confirming the smooth muscle origin of this benign tumor. In addition to these features, immunostaining is negative for CD117, the c-kit proto-oncogene, ruling out an extra-gastrointestinal stromal tumor and all of the cells are negative with S100, essentially excluding a tumor of neural origin.

Another tumor of the lung with features similar to a leiomyoma is the benign metastasizing leiomyoma. While this tumor is also a benign appearing smooth muscle tumor with an absence of atypia and mitotic count, benign metastasizing leiomyoma frequently presents as multiple lung lesions, commonly in a patient with a history of hysterectomy for benign uterine fibroids [6].

Treatment for all suspected leiomyomas of the lung whether symptomatic or not should be surgical resection for definitive diagnosis. In treating this entity, the surgical philosophy is to conserve tissue. Because of distal bronchiectasis and chronic inflammatory changes, lobectomy and pneumonectomy are common. For more peripheral tumor locations, a less radical resection is appropriate. Bronchoscopic resection is also possible, however the tumor is usually too extensive for this treatment [7].

The lesion must be distinguished from leiomyosarcoma. In contrast to leiomyoma, features of leiomyosarcoma include a well-differentiated smooth muscle lesion with an average mitotic count of 1/10 hpf, with or without increased cellularity or pleomorphism [5]. Prognosis for leiomyoma is generally excellent. There have been no reports of tumor recurrence following resection. The morbidity of the tumor is related to the morbidity of the operation.

## Conclusion

Benign pulmonary leiomyoma are rare tumors that are treated with conservative surgical resection. They are distinct from benign metastasizing leiomyoma and leiomyosarcoma. Following resection, they are associated with excellent prognosis.

## Abbreviations

CT: computerized tomography; SMA: smooth muscle actin.

## Consent

Written informed consent was obtained from the patient for publication of this case report and accompanying images. A copy of the written consent is available for review by the journal's Editor-in-Chief.

## Competing interests

The authors declare that they have no competing interests.

## Author's contributions

MV reviewed the case material and literature and was primary author. AK was the primary editor of this report. LP provided pathologic images and information on stains. PG was the primary pathologist analyzing the specimen. ML was the surgeon treating the patient. All authors read and approved the final manuscript.
